# Parenting behaviors and school burnout among Chinese primary school students: mediation model of ego-resilience

**DOI:** 10.3389/fpsyg.2026.1867806

**Published:** 2026-06-19

**Authors:** Yixin Sun, Mengxuan Wang, Xiang Li, Zhu Zhu, Yanlin Wang, Ruifeng Zhao, Yuko Sawada, Tokie Anme

**Affiliations:** 1 International Community Care and Lifespan Development: Empowerment Sciences, Doctoral Program in Medical Sciences, University of Tsukuba, Tsukuba, Ibaraki, Japan; 2 College of Child Development and Education, Zhejiang Normal University, Hangzhou, Zhejiang, China; 3 School of Public Health and Nursing, Hangzhou Normal University, Hangzhou, Zhejiang, China; 4 Department of Physical Therapy, Morinomiya University of Medical Sciences, Osaka, Japan; 5 Faculty of Medicine, University of Tsukuba, Tsukuba, Japan

**Keywords:** Chinese primary school students, ego-resilience, fathers’ and mothers’ parenting behaviors, mediation effect, school burnout

## Abstract

**Background:**

School burnout (SBO) poses a growing threat to the psychological well-being of primary school students in high-pressure educational systems. While negative parenting has been implicated in academic maladjustment, the psychological mechanisms underlying this association and whether they operate consistently across both parents remain poorly understood. This study investigates the mediating role of ego-resilience (ER) in the association between fathers’ and mothers’ negative parenting behaviors (rejection, overprotection, and lack of emotional warmth) and SBO among Chinese primary school students.

**Methods:**

This cross-sectional study was conducted in a public primary school in Hebei Province, China, involving 853 students from grades 4 through 6. Fathers’ and mothers’ parenting behaviors were assessed using the Chinese version of the Short-form Egna Minnen av. Barndoms Uppfostran (S-EMBU-C); SBO was evaluated using the Adolescent Student Burnout Inventory (ASBI), and ER was measured using the Chinese version of the Ego-Resilience Scale (ER89-C). Data were analyzed using Spearman’s rank correlation and mediation analysis via PROCESS macro (Model 4) to test the indirect effects of parenting behavior on SBO through ER.

**Results:**

Both fathers’ and mothers’ negative parenting was positively associated with SBO and negatively associated with ER. Mediation analyses revealed that ER significantly and partially mediated the relationship between negative parenting and SBO for both fathers (*β* = 0.16, 95% CI: [0.13, 0.20]) and mothers (*β* = 0.16, 95% CI: [0.13, 0.20]), accounting for 31.9 and 31.5% of the total effects, respectively.

**Conclusion:**

ER functions as a pivotal psychological mechanism through which both fathers’ and mothers’ negative parenting behaviors are associated with SBO. The structural consistency across both parents suggests that preserving students’ ER represents a universal protective target independent of parental role. These findings offer a theoretically grounded basis for family–school interventions aimed at fostering adaptive capacity and mitigating SBO in competitive educational contexts.

## 1 Introduction

School burnout (SBO) has become an increasingly important topic in psychological research, particularly because of its relevance to students’ academic adjustment and mental health. It refers to a psychological syndrome characterized by emotional exhaustion, cynicism, and a sense of inadequacy (Schaufeli et al., 2002). A recent review of SBO prevalence studies reported an average prevalence of 14.36% across 11 studies involving 18,016 students (Lacombe et al., 2023). SBO not only threatens academic performance and learning motivation (Madigan and Curran, 2021; Usán et al., 2022) but also increases the risk of long-term internalized problems such as anxiety and depression (Jiang et al., 2021; Liu et al., 2024; Song et al., 2024). These consequences highlight the importance of identifying risk and protective factors associated with SBO before burnout-related difficulties become more severe.

Although SBO has been more widely examined among college or secondary school students, emerging evidence suggests that burnout-related symptoms can also be observed during the primary school years (Gan, 2021; Yang et al., 2013). This issue is particularly important in the Chinese educational context, where academic achievement is strongly emphasized from an early age and is often regarded as a pathway to future educational opportunities and upward social mobility (Zhou et al., 2025). Chinese primary school students may therefore be exposed to intense academic competition, high parental expectations, and performance-oriented pressure while their coping strategies and self-regulatory capacities are still developing (Gao, 2023; Skinner and Zimmer-Gembeck, 2007; Morris et al., 2007). Therefore, examining SBO during the primary school period is important for identifying early signs of academic and psychosocial maladjustment and for informing timely prevention and intervention efforts.

The development of SBO is a complex process influenced by multiple systems. According to ecological systems theory (Bronfenbrenner, 1979), the family, as the core microsystem of socialization, has a profound and lasting impact on child development. In primary school education, the family environment—particularly parenting behavior—provides a critical context for coping with external pressures and shaping learning attitudes (Huang et al., 2021; Kim et al., 2024). In the current study, we conceptualized parenting behavior through three dimensions adapted from the Short-form Egna Minnen av. Barndoms Uppfostran: emotional warmth, rejection, and overprotection (Arrindell et al., 1999; Jiang et al., 2010). These dimensions have been widely used to assess perceived parenting behavior. Rejection reflects punitive, hostile, and dismissive parenting; emotional warmth reflects affectionate, supportive parenting; and overprotection reflects intrusive, highly controlling, and obedience-demanding parenting (Arrindell et al., 1998). Although the S-EMBU-C is structured around separate subscales, the present study constructed a study-specific composite index of “negative parenting behaviors” by reverse-coding emotional warmth and combining it with rejection and overprotection. A similar scoring approach has been used in prior research among Chinese primary school students, in which emotional warmth was reverse-coded and combined with rejection and overprotection to form an overall parenting style score (Yao et al., 2023). This operationalization was intended to capture the overall maladptive quality of parenting rather than the effect of any single parenting dimension. The association between parenting behavior and SBO can be understood through the conservation of resources (COR) theory (Hobfoll, 1989). From this perspective, SBO represents a state of psychological resource depletion. Negative parenting behavior—characterized by high rejection, high overprotection, and low emotional warmth—constitutes a chronic stressor within the family microsystem that is linked to continuous drainage of a child’s emotional reserves. When children perceive their parents as rejecting, emotionally unsupportive, or overly intrusive, they may be more likely to experience emotional exhaustion, cynicism, and reduced efficacy, which are central features of burnout (Lacombe et al., 2023; Li and Gan, 2011; Shin et al., 2012).

However, a critical limitation in existing research is the homogenization of parental roles. Fathers’ and mothers’ parenting behaviors are often combined or treated as functionally equivalent. However, this approach may obscure important differences. Although parenting behavior itself is the proximal factor influencing child development, similar parenting dimensions (such as rejection or overprotection) may carry different meanings and developmental implications depending on whether they are enacted by fathers or mothers. From a family systems perspective (Cox and Paley, 1997), father- and mother–child relationships represent distinct relational subsystems, within which children may differentially interpret and respond to parenting behaviors. Empirical evidence also highlights the importance of examining these parental roles separately. Whereas some studies have demonstrated that paternal acceptance has a significantly stronger association with children’s psychological adjustment than maternal acceptance (Khaleque and Rohner, 2012), other studies have reported the opposite pattern, with maternal rejection more strongly predicting psychological maladjustment (Ali et al., 2019); this suggests that the direction and magnitude of parental influence varies as a function of domain and outcome measures. In the academic domain in particular, maternal parenting behavior has been shown to exert a stronger influence on academic performance than paternal parenting behavior (Fute et al., 2024), while paternal rejection has been independently linked to motivational deficits and emotional disengagement (Khaleque and Rohner, 2012); these dimensions are conceptually central to SBO. These mixed and sometimes contradictory findings indicate that assuming homogeneity across parental roles is empirically untenable. Hence, we treated fathers’ and mothers’ parenting behaviors as analytically distinct variables, not merely to compare differences in gender but to determine whether and how similar parenting dimensions operate differentially across parental roles in predicting SBO.

Beyond establishing these distinct links, it is essential to explore the fundamental psychological pathways. Ego-resilience (ER), first conceptualized by Block and Block (1980) and operationalized by Block and Kremen (1996), refers to the ability to flexibly adapt to changing environmental demands and recover effectively from stress and adversity. Unlike general resilience, ER is a relatively stable yet developmentally malleable personality-level trait; it is shaped not merely by situational factors but by cumulative experiences within key developmental contexts, particularly the family (Alessandri et al., 2016; Farkas and Orosz, 2015). Empirically, individual differences in ER emerge early in childhood (Taylor et al., 2014) and have been linked to a range of adaptive outcomes, including emotion regulation, social competence (Milioni et al., 2014; Taylor et al., 2013a), and academic functioning (Swanson et al., 2011). The family environment plays a critical role in shaping children’s ER. Children exposed to adverse family circumstances (such as parental rejection or intrusive control) consistently show lower levels of ER compared to those raised in supportive contexts (Eisenberg et al., 2009; Taylor et al., 2013b). These characteristics make ER particularly relevant to the present study; as a family-sensitive internal resource, ER represents precisely the kind of psychological mechanism through which negative parenting behavior may shape children’s vulnerability to SBO.

Given the growing mental health burden among children worldwide, concern has deepened over the cultivation of ER. In China specifically, rapid socioeconomic changes, intensified academic competition, and high parental expectations have substantially elevated psychological stress among primary school students (Zhou et al., 2025). Current epidemiological data indicate that the prevalence of anxiety and depression symptoms among Chinese children and adolescents has risen markedly in recent years (Zhou et al., 2024), underscoring the urgent need to identify modifiable psychological resources that buffer children against academic stress. ER is particularly well-suited to serve this role; it functions as a core internal resource that enables children to cope with chronic stressors, and its sensitivity to family-based influences renders it a theoretically justified mediator through which parenting behavior may shape vulnerability to SBO.

Building on the conceptual foundation outlined above, we proposed ER as a central mediating variable linking parenting behavior to SBO. From a COR perspective (Hobfoll, 1989), negative parenting behavior constitutes a chronic resource-depleting stressor, and ER, as a key internal psychological resource, represents the mechanism through which this depletion translates into SBO.

Empirically, although few studies have directly explored the full dual-path mediation model, substantial evidence supports each link within this chain. Numerous studies have found a significant negative correlation between ER and SBO, confirming the former’s role as a protective factor against the latter (Gong et al., 2021; Shi et al., 2025). Furthermore, negative parenting behaviors have been identified as significant correlates of lower ER (Carroza-Pacheco et al., 2025; Moon et al., 2017; Ye et al., 2024; Yu et al., 2025). Taken together, these theoretical and empirical foundations suggest that negative parenting behavior may be linked to SBO by eroding a student’s personal psychological resources.

Despite growing attention to SBO, several important gaps remain. First, existing research has focused mainly on college or secondary school students, whereas SBO among primary school students remains relatively underexamined. This gap is important because the primary school period represents an earlier developmental stage in which academic pressure may interact with still-developing coping strategies and self-regulatory capacities. Second, fathers’ and mothers’ parenting behaviors are often combined or treated as functionally equivalent, which may obscure the distinct roles of paternal and maternal parenting. Third, the psychological mechanism linking negative parenting behaviors to SBO remains insufficiently understood. Particularly, few studies have examined whether ER serves as an internal adaptive resource through which fathers’ and mothers’ negative parenting behaviors are associated with SBO among Chinese primary school students. To address these gaps and examine the robustness of psychological mechanisms across different family subsystems, we employed separate mediation models for fathers and mothers to achieve the following objectives.

1. Examine the unique associations of fathers’ and mothers’ negative parenting behaviors with SBO among Chinese primary school students, respectively.
2. Clarify whether ER serves as a universal psychological mediator linking both fathers’ and mothers’ parenting behaviors to SBO.

We posited the following:

Hypothesis 1a (H1a): Fathers’ negative parenting behaviors are positively associated with SBO.

Hypothesis 1b (H1b): Mothers’ negative parenting behaviors are positively associated with SBO.

Hypothesis 2a (H2a): ER mediates the association between fathers’ negative parenting behaviors and SBO.

Hypothesis 2b (H2b): ER mediates the association between mothers’ negative parenting behaviors and SBO.

## 2 Methods

### 2.1 Study design

We employed a cross-sectional design. The data were gathered in 2024 using convenience sampling in a public primary school in Hebei Province, China. The inclusion criteria were: (1) students in grades 4 through 6; (2) voluntary participation from both the students and their caregivers; and (3) students residing with the participating caregiver. Exclusion criteria included students with previously diagnosed developmental or mental disorders that might interfere with their ability to provide accurate responses.

The recruitment process involved distributing an online questionnaire to eligible student-caregiver dyads through the school administration. Informed consent was obtained from all participating caregivers, and informed assent was obtained from students prior to data collection. The participants were informed that their involvement was entirely voluntary and that they could withdraw from the study at any time without any negative consequences. Only dyads where both the caregiver provided active consent and the student provided assent via the online platform were permitted to proceed to the survey. This study was approved by the Ethics Committee of Shanghai Sports University, China (102772023RT111) and the University of Tsukuba, Japan (1956).

### 2.2 Participants

A total of 878 dyads initially participated in the survey. Following systematic data matching and cleaning, we excluded 25 cases due to invalid responses (i.e., providing identical answers to all items). Consequently, the final analytical sample consisted of 853 valid student-caregiver dyads. Table 1 presents detailed demographic traits.

**Table 1:** Demographic characteristics of the study sample (*N* = 853).

Variables	Category	*n*	%
Grade	Grade 4	256	30.0
	Grade 5	272	31.9
	Grade 6	325	38.1
Sex	Boy	441	51.7
	Girl	412	48.3
Only-child status	Yes	236	27.7
	No	617	72.3
Family type	Two-parent family	802	94.0
	Single-parent and remarried family	51	6.0
Family structure	Nuclear family	624	73.2
	Extended family	229	26.8
Father’s education level	Below senior high school	159	18.6
	Senior high school and above	694	81.4
Mother’s education level	Below senior high school	158	18.5
	Senior high school and above	695	81.5
Annual family income	<60,000 CNY	210	24.6
	≥60,000 CNY	643	75.4

*N* = sample size; % = percentage of the total sample. CNY, Chinese yuan. The total sample size is 853, with all percentages calculated based on the full sample.

### 2.3 Measure

#### 2.3.1 School burnout

Students assessed SBO using the Adolescent Student Burnout Inventory (ASBI; Wu et al., 2010), a 16-item self-report scale measuring three dimensions: exhaustion (4 items), academic cynicism (5 items), and reduced efficacy (7 items). Items are rated on a 5-point Likert scale ranging from 1 (*strongly inconsistent*) to 5 (*strongly consistent*). To calculate the composite SBO score, we reverse-coded some items, with higher total scores denoting greater levels of burnout. In the current study, the scale demonstrated excellent internal consistency (Cronbach’s *α* = 0.90).

#### 2.3.2 Parenting behaviors

Parenting behaviors were assessed using the Chinese version of the Short-form Egna Minnen av. Barndoms Uppfostran (S-EMBU-C; Jiang et al., 2010), adapted from the original S-EMBU (Arrindell et al., 1999). The S-EMBU-C is a 21-item self-report scale measuring three dimensions: rejection (6 items), emotional warmth (7 items), and overprotection (8 items). Students rated their fathers’ and mothers’ parenting behaviors separately on a 4-point Likert scale (1 = never, 4 = always). To represent the overall maladaptive quality of parenting, emotional warmth items were reverse-coded and then combined with rejection and overprotection items to calculate a composite negative parenting score. This study-specific composite score was constructed based on the three core S-EMBU-C dimensions and was used to examine the general resource-depleting quality of father’s and mother’s parenting behaviors. Higher total scores indicate greater levels of negative parenting. In the current study, Cronbach’s *α* for both the paternal and maternal total scales was 0.84, suggesting good internal consistency.

#### 2.3.3 Ego-resilience

Students assessed ER using the Chinese Version of the Ego-Resilience Scale (ER89-C; Chen et al., 2019), adapted from Block and Kremen’s (1996) ER89. This self-report scale consists of 14 items rated on a 4-point Likert scale (1 = *does not apply at all*, 4 = *applies very strongly*). The total score is calculated as the sum of each item. Higher scores indicate greater levels of ER. In this study, the scale showed good internal consistency (Cronbach’s α = 0.87).

#### 2.3.4 Covariates

Based on prior research and theoretical considerations, we controlled for several sociodemographic variables in the mediation models to account for potential confounding effects. These included students’ grade, sex, only-child status, family structure (nuclear vs. extended family), family type (two-parent family vs. single-parent or remarried family), father’s and mother’s education levels, and annual family income.

### 2.4 Statistical analysis

We analyzed the data using IBM SPSS Statistics (Version 26.0) and the PROCESS macro (Version 4.2). Given the online survey format where all items were mandatory, no missing data were present.

First, we conducted Harman’s single-factor test to examine potential common method bias (CMB). Second, we performed descriptive statistics and Spearman’s rank correlation analyses to explore the preliminary relationships among the variables. Third, we tested the mediation models using PROCESS Model 4. To assess the significance of the indirect effects, we employed bootstrapping with 5,000 resamples to generate a 95% bias-corrected confidence interval (CI). We deemed an indirect effect to be significant if the 95% CI did not include zero. We dummy-coded sociodemographic variables and included them as covariates in the models.

## 3 Results

### 3.1 Testing for common method bias

To examine potential CMB, we carried out Harman’s single-factor test using all items of the variables. The results of the unrotated exploratory factor analysis showed that the first principal factor explained 22.70% of the total variance, which is below the critical threshold of 40%, suggesting that no single factor accounted for the majority of the variance. This indicates that CMB was not a significant concern in the current study.

### 3.2 Sample characteristics

The final analytic sample consisted of 853 students from grades 4 through 6. Approximately 30.0% of the participants were in Grade 4, 31.9% in Grade 5, and 38.1% in Grade 6. Boys accounted for 51.7% of the sample, and 27.7% of the students were only children. Most of the participants lived in two-parent families (94.0%) and nuclear families (73.2%). As for parents’ education level, over 80.0% of fathers (81.4%) and mothers (81.5%) had graduated from senior high school or above. The majority of families reported an annual household income of 60,000 CNY or higher (75.4%). Table 1 displays detailed demographic information.

### 3.3 Descriptive statistics and correlation analysis

Table 2 portrays the descriptive statistics, correlations, and comparisons of the dimensions of fathers’ and mothers’ parenting behaviors. Wilcoxon signed-rank tests revealed significant differences between fathers’ and mothers’ parenting behaviors across all three dimensions (*p* < 0.05). Specifically, mothers were perceived to exhibit higher levels of rejection and overprotection, while fathers were viewed as showing lower emotional warmth.

**Table 2:** Descriptive statistics, correlations, and comparisons of the dimensions of fathers’ and mothers’ parenting behaviors.

Variable	Father M (SD)	Mother M (SD)	M (SD)	*z*	1	2	3	4	5	6	7	8
1. Father’s rejection	1.38 (0.45)				—							
2. Father’s low warmth	1.74 (0.59)				0.49**	—						
3. Father’s overprotection	1.96 (0.46)				0.49**	0.15**	—					
4. Mother’s rejection		1.40 (0.45)		−2.72**	0.73**	0.42**	0.40**	—				
5. Mother’s low warmth		1.60 (0.53)		−11.86**	0.42**	0.81**	0.18**	0.48**	—			
6. Mother’s overprotection		2.08 (0.48)		−12.45**	0.43**	0.21**	0.81**	0.50**	0.24**	—		
7. School burnout			1.96 (0.66)		0.45**	0.53**	0.21**	0.46**	0.51**	0.28**	—	
8. Ego-resilience			3.12 (0.47)		−0.29**	−0.52**	−0.14**	−0.29**	−0.55**	−0.17**	−0.58**	—

*N* = 853. Values are presented as means (standard deviations). r_s denotes Spearman’s rank-order correlation coefficients. Wilcoxon signed-rank tests were used to compare the dimensions of fathers’ and mothers’ parenting behaviors; z values represent standardized test statistics. For the dimensions of negative parenting (rejection, low warmth, and overprotection), higher scores indicate greater levels of negative parenting behaviors. For ego-resilience and school burnout, higher scores indicate greater levels of the respective constructs. **p < 0.01, ***p < 0.001.

The correlation analysis demonstrated that all dimensions of negative parenting behaviors for both fathers and mothers were significantly positively associated with SBO and negatively associated with ER. The consistent direction of these correlations across rejection, low warmth, and overprotection supported their use as indicators of an overall maladptive parenting construct in the main mediation analyses. ER was strongly negatively correlated with SBO (*r* = −0.58, *p* < 0.01).

### 3.4 Mediation analyses

To test the mediating role of ER in the relationship between negative parenting behaviors and SBO, we conducted mediation analyses using Model 4 of the PROCESS macro. We tested separate models for fathers’ and mothers’ negative parenting, with the results outlined in Table 3. All analyses controlled for students’ grade, sex, only-child status, family structure, family type, father’s and mother’s education levels, and annual family income.

**Table 3:** Mediation analyses of ego-resilience in the relationship between fathers’ and mothers’ negative parenting and school burnout.

Model & Path	*β*	*SE*	*t*	*p*	95% CI [LL, UL]	Effect ratio
Panel A: Fathers’ model						
Path a (FNP → ER)	−0.39	0.04	−12.40	<0.001	[−0.57, −0.41]	—
Path b (ER → SBO)	−0.42	0.04	−14.58	<0.001	[−0.66, −0.50]	—
Direct effect (c’)	0.35	0.05	12.22	<0.001	[0.51, 0.71]	68.1%
Indirect effect (ab)	0.16	0.02	—	—	[0.13, 0.20]	31.9%
Total effect (c)	0.51	0.05	17.44	<0.001	[0.80, 1.00]	100.0%
Panel B: Mothers’ model						
Path a (MNP → ER)	−0.40	0.04	−12.92	<0.001	[−0.59, −0.43]	—
Path b (ER → SBO)	−0.41	0.04	−14.23	<0.001	[−0.65, −0.49]	—
Direct effect (c’)	0.36	0.05	12.58	<0.001	[0.53, 0.73]	68.5%
Indirect effect (ab)	0.16	0.02	—	—	[0.13, 0.20]	31.5%
Total effect (c)	0.52	0.05	18.06	<0.001	[0.82, 1.02]	100.0%

β, completely standardized coefficients; SE, standard error; CI, confidence interval; LL, lower limit; UL, upper limit. FNP, Fathers’ negative parenting; MNP, mothers’ negative parenting; ER, ego-resilience; SBO, school burnout. Effect ratio = (indirect effect/total effect) × 100. The 95% confidence interval (CI) for indirect effects was estimated using 5,000 bootstrap resamples. Covariates (grade, sex, only-child status, family structure, family type, father’s and mother’s education levels, and family income) were controlled for in all models. *N* = 853.

#### 3.4.1 Fathers’ negative parenting model

As seen in Table 3 (Panel A), fathers’ negative parenting (FNP) was significantly and negatively associated with ER (path a: *β* = −0.39, *SE* = 0.04, *t* = −12.40, *p* < 0.001). ER, in turn, was significantly and negatively linked to SBO (path b: *β* = −0.42, *SE* = 0.04, *t* = −14.58, *p* < 0.001). The direct relationship between FNP and SBO remained significant (path c’: *β* = 0.35, *SE* = 0.05, *t* = 12.22, *p* < 0.001). The indirect path from FNP to SBO via ER was significant (*β* = 0.16, *BootSE* = 0.02, *95%CI* [0.13, 0.20]) as the bootstrap 95% CI did not include zero. This mediating path accounted for 31.9% of the total relationship (*β* = 0.51, *p* < 0.001).

#### 3.4.2 Mothers’ negative parenting model

Similarly, in the mothers’ model (Panel B), mothers’ negative parenting (MNP) was significantly and negatively related to ER (path a: *β* = −0.40, *SE* = 0.04, *t* = −12.92, *p* < 0.001). ER was significantly and negatively associated with SBO (path b: *β* = −0.41, *SE* = 0.04, *t* = −14.23, *p* < 0.001). The direct relationship between MNP and SBO remained significant (path c’: *β* = 0.36, *SE* = 0.05, *t* = 12.58, *p* < 0.001). The indirect effect via ER was significant (*β* = 0.16, *BootSE* = 0.02, *95%CI* [0.13, 0.20]), suggesting that ER partially mediated the relationship between MNP and SBO. The mediation path accounted for 31.5% of the total relationship (*β* = 0.52, *p* < 0.001).

## 4 Discussion

We investigated the association between fathers’ and mothers’ negative parenting and SBO among Chinese primary school students, focusing on the mediating role of ER. Both fathers’ and mothers’ negative parenting behaviors were significantly associated with increased SBO, and these relationships were partially mediated by ER. These findings suggest that family-related experiences and children’s internal adaptive resources are both relevant to understanding SBO vulnerability during the primary school years. Accordingly, the following discussion focuses on how negative parenting behaviors and ER help explain SBO among Chinese primary school students.

The direct associations between fathers’ and mothers’ negative parenting behaviors and higher SBO indicate that parenting experiences are closely related to children’s academic adjustment. This finding is consistent with prior studies linking parenting behaviors to SBO. Among medical students, Teng et al. (2025) found that rejection and overprotection were positively associated with SBO, whereas emotional warmth was negatively associated with SBO. Additionally, Zhu et al. (2023) showed that maternal and paternal parenting behaviors were related to adolescents’ academic engagement and burnout. Extending this evidence to primary school students, the present study suggests that the overall maladaptive quality of fathers’ and mothers’ parenting behaviors is associated with SBO at an earlier developmental stage. Therefore, SBO among primary school students may be better understood not only as a response to academic demands but also as an academic maladjustment associated with family relational experiences.

The negative associations between fathers’ and mothers’ negative parenting behaviors and ER further suggest that parenting experiences are closely related to children’s internal adaptive resources. From the ecological systems perspective, negative parenting behaviors, such as rejection, insufficient emotional warmth, and overprotection, may be associated with a stressful family relational climate and weaker self-regulatory resources and recovery capacity (Khurana et al., 2024; Cai et al., 2022; Ding et al., 2023).

The strong negative association between ER and SBO also deserves specific attention. In the correlation analysis, ER showed a strong negative association with SBO (*r* = −0.58), and ER remained significantly associated with SBO in the mediation models. This pattern suggests that ER may be a particularly important internal resource for understanding SBO vulnerability among Chinese primary school students. In the demanding Chinese academic context, characterized by high evaluative pressure and social comparison (Ding et al., 2022), the capacity to flexibly adapt to setbacks and sustain engagement may be especially relevant to whether academic demands are experienced as manageable challenges or as chronic stressors. From the perspective of COR theory (Hobfoll and Freedy, 1993), ER may help students preserve psychological resources when facing repeated academic demands, thereby reducing vulnerability to the exhaustion, cynicism, and reduced efficacy that characterize SBO (Jiang and Gao, 2025; Vansoeterstede et al., 2023).

The mediation models further indicate that negative parenting behaviors are associated with SBO partly through lower ER, with this indirect pathway accounting for approximately 31.5% to 31.9% of the total association. This finding is consistent with the loss spiral principle of COR theory (Hobfoll et al., 2018), whereby an initial reduction in personal resources may increase vulnerability to further resource depletion. In the present study, negative parenting behaviors may represent a family-based stressor associated with lower internal adaptive resources, which in turn are linked to greater burnout symptoms in the school context. This process can also be understood as a cross-contextual spillover effect (Infante-Cañete et al., 2022): psychological strain originating in the family microsystem may be carried into academic life through weakened ER. In this sense, ER helps explain how family relational experiences may become associated with SBO in the academic context.

Interestingly, mothers were perceived to exhibit significantly higher levels of rejection and overprotection than fathers. Although findings on gender differences in parenting are mixed in the existing literature (e.g., Dou et al., 2020; Guo and Feng, 2017), this result is consistent with evidence from Chinese samples showing higher perceived maternal control and rejection relative to fathers (Xu et al., 2024). It may also be understood in light of China’s contemporary “intensive mothering” culture, in which mothers often bear a disproportionate share of childcare responsibilities and emotional labor (Chen and Hou, 2024; Hanser and Li, 2017). However, the mediation mechanism appeared to function consistently across fathers’ and mothers’ parenting. This suggests that, although fathers and mothers may differ in the level or form of negative parenting behaviors, lower ER may represent a common pathway through which negative parenting behaviors are associated with greater vulnerability to SBO. Therefore, prevention and intervention efforts should consider the contributions of both parents rather than focusing exclusively on either fathers’ or mothers’ parenting.

These findings are meaningful given the developmental stage and educational context of Chinese primary school students. At this stage, children may experience increasing academic pressure while their coping strategies and self-regulatory capacities are still developing. The present findings suggest that SBO may already be relevant during the primary school years and that negative parenting behaviors and lower ER may be associated with greater burnout vulnerability. This highlights the need for early identification and prevention before burnout-related difficulties become more persistent.

These findings have significant practical implications for both family-level interventions and school mental health programs. At the family level, school counselors and guidance teachers could develop structured psycho-education programs for parents to increase awareness of the potential negative consequences of rejection, insufficient emotional warmth and overprotection. Such programs may include parent workshops, case-based discussions, and communication-skills training aimed at reducing overprotective practices and promoting more autonomy-supportive and emotionally responsive parenting. At the student level, schools could organize group counseling programs based on cognitive-behavioral and emotion-regulation approaches to strengthen students’ ER, such as cognitive reframing, problem-solving training, emotional awareness exercises and adaptive coping practices. By targeting both parenting practices and students’ internal coping resources, such operational family–school collaboration may help reduce children’s vulnerability to SBO in high-pressure educational contexts.

Despite its contributions, this study has several limitations that suggest avenues for future research. First, the cross-sectional design limits the ability to draw causal conclusions, particularly regarding the mediation model. Although the proposed model was theoretically grounded, the temporal ordering among negative parenting behaviors, ER, and SBO could not be definitively established. Therefore, the indirect associations observed in this study should not be interpreted as evidence of causality. Future longitudinal studies are needed to verify the directionality of these associations and to examine whether ER mediates the prospective relationship between negative parenting behaviors and SBO. Second, the reliance on self-report questionnaires may introduce social desirability bias or common method variance, suggesting that future research would benefit from a multi-informant approach incorporating teacher or parent reports. Third, the use of a composite negative parenting score is a limitation. Although this score captured the overall maladaptive quality of parenting, combining rejection, low emotional warmth, and overprotection may have obscured dimension-specific effects. Future studies should examine these dimensions separately or use parallel mediation models to clarify their distinct pathways to ER and SBO. Finally, the sample was restricted to a single urban area in China, which may limit the generalizability of the findings to more diverse socio-economic or rural contexts.

## 5 Conclusion

In sum, we identified ER as a consistent mediator linking both fathers’ and mothers’ negative parenting behaviors (rejection, overprotection, and lack of emotional warmth) to SBO among Chinese primary school students. These findings extend COR theory to the family–school interface, suggesting that the overall maladaptive quality of parenting is associated with students’ internal adaptive capacity and SBO. Practically, the findings suggest that school counselors and guidance teachers may translate family–school collaboration into concrete support through parent-focused psycho-education to reduce overprotective and rejecting parenting practices and student group counseling based on cognitive-behavioral or emotion-regulation strategies to strengthen ER.

## Data Availability

The raw data supporting the conclusions of this article will be made available by the authors, without undue reservation.

## References

[ref1] Ali S. Khatun N. Khaleque A. Rohner R. P. (2019). They love me not: a meta-analysis of relations between parental undifferentiated rejection and offspring’s psychological maladjustment. J. Cross-Cult. Psychol. 50, 185–199. doi: 10.1177/0022022118815599

[ref111] Alessandri G. Eisenberg N. Vecchione M. Caprara G. V. Milioni M. (2016). Ego-resiliency development from late adolescence to emerging adulthood: A ten-year longitudinal study. J. Adolesc. 50, 91–102. doi: 10.1016/j.adolescence.2016.05.004 27236209

[ref2] Arrindell W. A. Gerlsma C. Vandereycken W. Hageman W. J. J. M. Daeseleire T. (1998). Convergent validity of the dimensions underlying the parental bonding instrument (PBI) and the EMBU. Pers. Individ. Dif. 24, 341–350. doi: 10.1016/S0191-8869(97)00187-6

[ref3] Arrindell W. A. Sanavio E. Aguilar G. Sica C. Hatzichristou C. Eisemann M. . (1999). The development of a short form of the EMBU: its appraisal with students in Greece, Guatemala, Hungary and Italy. Pers. Individ. Differ. 27, 613–628. doi: 10.1016/S0191-8869(98)00192-5

[ref4] Block J. H. Block J. (1980). “The role of ego-control and ego-resiliency in the organization of behavior,” in Development of Cognition, Affect and Social Relations: The Minnesota Symposia on Child Psychology, ed. Collins W. A. (Hillsdale: Erlbaum).

[ref410] Block J. Kremen A. M. (1996). IQ and ego-resiliency: Conceptual and empirical connections and separateness. J. Pers. Soc. Psychol. 70, 349–361. doi: 10.1037/0022-3514.70.2.349 8636887

[ref5] Bronfenbrenner U. (1979). The Ecology of Human Development: Experiments by Nature and Design. Cambridge: Harvard University Press.

[ref6] Cai X. B. Gui S. C. Tang Y. Q. Zhang S. Xu M. S. (2022). The impact of parenting styles on resilience of adolescent: the chain mediating effect of self-control and regulatory focus. Psychol. Dev. Educ. 38, 505–512. doi: 10.16187/j.cnki.issn1001-4918.2022.04.06

[ref7] Carroza-Pacheco A. M. Bringas-Molleda C. León-del-Barco B. (2025). Parenting styles and key aspects of resilience in secondary education students. Adolescents. 5:75. doi: 10.3390/adolescents5040075 PMC12113049 40422656

[ref8] Chen X. He J. Fan X. (2019). Applicability of the ego-resilience scale (ER89) in the Chinese cultural context: a validation study. J. Psychoeduc. Assess. 38, 675–691. doi: 10.1177/0734282919889242

[ref9] Chen S. Hou Y. (2024). Digitally intensive mothering and unequal emotional work of Chinese mothers. Camb. J. Educ. 54, 663–679. doi: 10.1080/0305764X.2024.2405669

[ref10] Cox M. J. Paley B. (1997). Families as systems. Annu. Rev. Psychol. 48, 243–267. doi: 10.1146/annurev.psych.48.1.243 9046561

[ref11] Ding Z. E. Liu R. D. Ding Y. Yang X. T. Hong W. Li H. Z. (2022). The concurrent and longitudinal relations between competitive classroom climate and learning motivation among Chinese adolescent students: the mediating roles of social comparisons. Psychol. Res. Behav. Manag. 15, 1209–1219. doi: 10.2147/PRBM.S364803, 35592765 PMC9111041

[ref12] Ding X. Zheng L. Liu Y. T. Zhang W. Y. Wang N. Y. Duan H. X. . (2023). Parenting styles and psychological resilience: the mediating role of error monitoring. Biol. Psychol. 180:108587. doi: 10.1016/j.biopsycho.2023.108587 37224937

[ref13] Dou D. Shek D. T. L. Kwok K. H. R. (2020). Perceived paternal and maternal parenting attributes among Chinese adolescents: a meta-analysis. Int. J. Environ. Res. Public Health 17:8741. doi: 10.3390/ijerph17238741, 33255504 PMC7727811

[ref14] Eisenberg N. Chang L. Ma Y. Huang X. (2009). Relations of parenting style to Chinese children’s effortful control, ego resilience, and maladjustment. Dev. Psychopathol. 21, 455–477. doi: 10.1017/S095457940900025X, 19338693 PMC2771550

[ref15] Farkas D. Orosz G. (2015). Ego-resiliency reloaded: a three-component model of general resiliency. PLoS One 10:e0120883. doi: 10.1371/journal.pone.0120883, 25815881 PMC4376776

[ref16] Fute A. Oubibi M. Sun B. Zhou Y. Bassiri M. Chen G. (2024). Parenting for success: exploring the link between parenting styles and adolescents’ academic achievement through their learning engagement. SAGE Open 14:2. doi: 10.1177/21582440241255176

[ref17] Gan C. (2021). Investigation on the Current Situation of Academic Burnout of Elementary School Students in rural Areas: Taking the High Grade Elementary School Students in Z City as an Example [Master’s thesis]. Jining: Qufu Normal University.

[ref18] Gao X. (2023). Academic stress and academic burnout in adolescents: a moderated mediating model. Front. Psychol. 14:1133706. doi: 10.3389/fpsyg.2023.1133706, 37342640 PMC10278958

[ref19] Gong Z. Li C. Jiao X. Qu Q. (2021). Does resilience help in reducing burnout symptoms among Chinese students? A meta-analysis. Front. Psychol. 12:707792. doi: 10.3389/fpsyg.2021.707792, 34489811 PMC8418100

[ref20] Guo Q. Feng L. (2017). The associations between perceived parenting styles, empathy, and altruistic choices in economic games: a study of Chinese children. Front. Psychol. 8:1843. doi: 10.3389/fpsyg.2017.01843, 29085324 PMC5650632

[ref21] Hanser A. Li J. (2017). The hard work of feeding the baby: breastfeeding and intensive mothering in contemporary urban China. J. Chin. Sociol. 4:18. doi: 10.1186/s40711-017-0065-2

[ref23] Hobfoll S. E. (1989). Conservation of resources: a new attempt at conceptualizing stress. Am. Psychol. 44, 513–524. doi: 10.1037//0003-066x.44.3.513, 2648906

[ref24] Hobfoll S. E. Freedy J. (1993). “Conservation of resources: a general stress theory applied to burnout,” in Professional Burnout: Recent Developments in Theory and Research, eds. Schaufeli W. B. Maslach C. Marek T. (London: Taylor & Francis), 115–133.

[ref25] Hobfoll S. E. Halbesleben J. Neveu J.-P. Westman M. (2018). Conservation of resources in the organizational context: the reality of resources and their consequences. Annu. Rev. Organ. Psychol. Organ. Behav. 5, 103–128. doi: 10.1146/annurev-orgpsych-032117-104640

[ref26] Huang X. Hua L. Zhou X. Zhang H. Zhang M. Wang S. . (2021). The association between home environment and quality of life in children and adolescents in Hangzhou City, China. J. Child Fam. Stud. 30, 1416–1427. doi: 10.1007/s10826-021-01951-1

[ref27] Infante-Cañete L. Arias-Calero L. Wallace-Ruiz A. Sánchez-Sánchez A. M. Muñoz-Sánchez Á. (2022). One more step in the study of children’s daily stress: the spillover effect as the transfer of tension in family and school environments. Front. Psychol. 13:909928. doi: 10.3389/fpsyg.2022.909928, 36571012 PMC9768336

[ref28] Jiang C. C. Gao Q. F. (2025). Exploring resilience mechanism in learning burnout among pupils: school adjustment and academic self-efficacy. Front. Psychol. 16:1706567. doi: 10.3389/fpsyg.2025.1706567, 41383425 PMC12691449

[ref29] Jiang J. Lu Z. Jiang B. Xu Y. (2010). Revision of the short-form Egna Minnenav barndoms uppfostran for Chinese. Psychol. Dev. Educ. 26, 94–99. doi: 10.16187/j.cnki.issn1001-4918.2010.01.017

[ref30] Jiang S. Ren Q. Jiang C. Wang L. (2021). Academic stress and depression of Chinese adolescents in junior high schools: moderated mediation model of school burnout and self-esteem. J. Affect. Disord. 295, 384–389. doi: 10.1016/j.jad.2021.08.085, 34492431

[ref31] Khaleque A. Rohner R. P. (2012). Pancultural associations between perceived parental acceptance and psychological adjustment of children and adults: a meta-analytic review of worldwide research. J. Cross-Cult. Psychol. 43, 784–800. doi: 10.1177/0022022111406120

[ref32] Khurana A. Leonard H. Michaelson L. Kosty D. (2024). Transactional linkages between parenting behaviors and child executive functions and self-regulation from early childhood to adolescence. J. Pers. Oriented Res. 10, 26–55. doi: 10.17505/jpor.2024.26261, 38841560 PMC11149413

[ref33] Kim T. De Gruiter M. Kuppens L. (2024). Psychosocial impact of adverse home environment on primary school performance in Cambodia. Int. J. Prof. Dev. Learn. Learn. 6:e2410. doi: 10.30935/ijpdll/15172, 42135485

[ref34] Lacombe N. Hey M. Hofmann V. Pagnotta C. Squillaci M. (2023). School burnout after COVID-19, prevalence and role of different risk and protective factors in preteen students. Children (Basel). 10:823. doi: 10.3390/children10050823, 37238371 PMC10217600

[ref35] Li M. Gan Y. (2011). Relationship between academic burnout tendency and parenting styles among high school students. Chin. J. Sch. Health. 32, 36–37. doi: 10.16835/j.cnki.1000-9817.2011.01.017

[ref36] Liu W. Zhang R. Wang H. Rule A. Wang M. Abbey C. . (2024). Association between anxiety, depression symptoms, and academic burnout among Chinese students: the mediating role of resilience and self-efficacy. BMC Psychol. 12:335. doi: 10.1186/s40359-024-01823-5, 38849921 PMC11162059

[ref37] Madigan D. J. Curran T. (2021). Does burnout affect academic achievement? A meta-analysis of over 100,000 students. Educ. Psychol. Rev. 33, 387–405. doi: 10.1007/s10648-020-09533-1

[ref38] Milioni M. Alessandri G. Eisenberg N. Castellani V. Zuffianò A. Vecchione M. . (2014). Reciprocal relations between emotional self-efficacy beliefs and ego-resiliency across time. J. Pers. 83, 552–563. doi: 10.1111/jopy.12131, 25204666 PMC4709220

[ref39] Moon J. R. Song J. Huh J. Kang I. S. Park S. W. Chang S. A. . (2017). The relationship between parental rearing behavior, resilience, and depressive symptoms in adolescents with congenital heart disease. Front. Cardiovasc. Med. 4:55. doi: 10.3389/fcvm.2017.00055, 28944224 PMC5596069

[ref40] Morris A. S. Silk J. S. Steinberg L. Myers S. S. Robinson L. R. (2007). The role of the family context in the development of emotion regulation. Soc. Dev. 16, 361–388. doi: 10.1111/j.1467-9507.2007.00389.x, 19756175 PMC2743505

[ref41] Schaufeli W. B. Salanova M. González-romá V. Bakker A. B. (2002). The measurement of engagement and burnout: a two sample confirmatory factor analytic approach. J. Happiness Stud. 3, 71–92. doi: 10.1023/A:1015630930326

[ref42] Shi Y. Zhao W. Lipowski M. (2025). Effects of physical activity and resilience on emotional and behavioral problems in Chinese adolescent: a chained mediation model. Front. Psychol. 16:1486949. doi: 10.3389/fpsyg.2025.1486949, 40693157 PMC12277388

[ref43] Shin H. Lee J. Kim B. Lee S. M. (2012). Students’ perceptions of parental bonding styles and their academic burnout. Asia Pacific Educ. Rev. 13, 509–517. doi: 10.1007/s12564-012-9218-9

[ref44] Skinner E. A. Zimmer-Gembeck M. J. (2007). The development of coping. Annu. Rev. Psychol. 58, 119–144. doi: 10.1146/annurev.psych.58.110405.085705 16903804

[ref45] Song S. Guo R. Chen X. Li C. (2024). How school burnout affects depression symptoms among Chinese adolescents: evidence from individual and peer clique level. J. Youth Adolesc. 54, 92–102. doi: 10.1007/s10964-024-02044-0 38965138

[ref46] Swanson J. Valiente C. Lemery-Chalfant K. Caitlin O’Brien T. (2011). Predicting early adolescents’ academic achievement, social competence, and physical health from parenting, ego resilience, and engagement coping. J. Early Adolesc. 31, 548–576. doi: 10.1177/027243161036624

[ref47] Taylor Z. E. Eisenberg N. Spinrad T. L. Widaman K. F. (2013b). Longitudinal relations of intrusive parenting and effortful control to ego-resiliency during early childhood. Child Dev. 84, 1145–1151. doi: 10.1111/cdev.12054, 23379965 PMC3659177

[ref48] Taylor Z. E. Eisenberg N. VanSchyndel S. K. Eggum-Wilkens N. D. Spinrad T. L. (2013a). Children’s negative emotions and ego-resiliency: longitudinal relations with social competence. Emotion 14, 397–406. doi: 10.1037/a0035079, 24364850 PMC4472430

[ref49] Taylor Z. E. Sulik M. J. Eisenberg N. Spinrad T. L. Silva K. M. Lemery-Chalfant K. L. . (2014). Development of ego-resiliency: relations to observed parenting and polymorphisms in the serotonin transporter gene during early childhood. Soc. Dev. 23, 433–450. doi: 10.1111/sode.12041, 25346579 PMC4206910

[ref50] Teng C. Shi L. Xu C. Zhu J. (2025). Parenting styles and academic burnout in medical students: a moderated mediation model of resilience and stress. Front. Med. (Lausanne). 12:1703534. doi: 10.3389/fmed.2025.1703534, 41293719 PMC12640909

[ref51] Usán P. Salavera C. Quílez-Robres A. Lozano-Blasco R. (2022). Behaviour patterns between academic motivation, burnout and academic performance in primary school students. Int. J. Environ. Res. Public Health 19:12663. doi: 10.3390/ijerph191912663, 36231963 PMC9566615

[ref52] Vansoeterstede A. Cappe E. Lichtlé J. Boujut E. (2023). A systematic review of longitudinal changes in school burnout among adolescents: trajectories, predictors, and outcomes. J. Adolesc. 95, 224–247. doi: 10.1002/jad.12121, 36385709

[ref53] Wu Y. Dai X. Wen Z. Cui H. (2010). Development of adolescents’ school burnout scale. Chin. J. Clin. Psychol. 18:2. doi: 10.16128/j.cnki.1005-3611.2010.02.018

[ref54] Xu X. Hanafi Z. Razak N. A. (2024). Exploring sociodemographic correlates of fathers’ and mothers’ behavioral control. Behav. Sci. (Basel). 14:1203. doi: 10.3390/bs14121203, 39767344 PMC11673098

[ref55] Yang L. Gao Z. Wang S. Li H. Han Z. Zhang Y. (2013). Learning burnout of pupils in grade 4 to 6 in Tangshan city. China J. Health Psychol. 21, 442–444. doi: 10.13342/j.cnki.cjhp.2013.03.025

[ref56] Yao X. Zhang Y. Wang J. (2023). Analysis of the effects of parenting styles on learning burnout and the mediating effect of peer relationships among primary school students based on SPSS. Adv. Hum. Cult. Stud. 5, 143–154. doi: 10.2991/978-94-6463-034-3_16

[ref57] Ye S. Jenatabadi H. S. Ye M. Chen M. Lin X. Mustafa Z. (2024). Academic resilience, self-efficacy, and motivation: the role of parenting style. Sci. Rep. 14:5571. doi: 10.1038/s41598-024-55530-7, 38448465 PMC10918079

[ref58] Yu M. Tng G. Y. Q. Lin Z. Chen H. Eriksson J. G. Chong Y. S. . (2025). Early socioeconomic conditions to children’s trait resilience: longitudinal mediation effects of mothers’ and fathers’ parenting. Child Adolesc. Psychiatry Ment. Health 19:123. doi: 10.1186/s13034-025-00979-1, 41214755 PMC12604427

[ref59] Zhou J. Liu Y. Ma J. Feng Z. Hu J. Hu J. . (2024). Prevalence of depressive symptoms among children and adolescents in China: a systematic review and meta-analysis. Child Adolesc. Psychiatry Ment. Health 18:150. doi: 10.1186/s13034-024-00841-w, 39563377 PMC11577650

[ref60] Zhou J. Tang W. Sun Z. Xue S. Liu Q. Wu S. . (2025). Parental expectation and psychological distress of Chinese youth: the chain mediating effects of core self-worth and perceived stress. BMC Public Health 25:3457. doi: 10.1186/s12889-025-24759-w, 41074014 PMC12512374

[ref61] Zhu Q. Cheong Y. Wang C. Tong J. (2023). The impact of maternal and paternal parenting styles and parental involvement on Chinese adolescents’ academic engagement and burnout. Curr. Psychol. 42, 2827–2840. doi: 10.1007/s12144-021-01611-z

